# Can Physical Exercise Help Deal With the COVID-19 Stressors? Comparing Somatic and Psychological Responses

**DOI:** 10.3389/fpsyg.2022.883801

**Published:** 2022-07-13

**Authors:** Junwei Qian, Jiajin Tong, Ruiheng Xu

**Affiliations:** ^1^Department of Physical Education and Research, Peking University, Beijing, China; ^2^Beijing Key Laboratory of Behavior and Mental Health, School of Psychological and Cognitive Sciences, Peking University, Beijing, China

**Keywords:** physical exercise, COVID-19 stressors, somatic complaints, worry complaints, sleep disorder, cross-stressor adaptation hypothesis

## Abstract

This research aims to explore whether physical exercise can buffer the impact of the COVID-19 stressors. Based on the cross-stressor adaptation hypothesis, we proposed a moderated mediation model relating the COVID-19 stressors to sleep disorder *via* somatic and worry complaints, depending on the amount of physical exercise. A sample of working adults in Beijing (*N* = 207) filled surveys in two waves during the COVID-19 pandemic. Structural regression analysis showed that physical exercise moderates the impact of the COVID-19 stressors on sleep disorder via somatic complaints (*index* = −0.11, 95% CI [−0.22, −0.01]), rather than psychological worry complaints (*index* = −0.01, 95% *CI* [−0.07, 0.04]). Specifically, the COVID-19 stressors increase somatic complaints for people with a low amount of physical exercise (*b* = 0.17, *p* = 0.01]), while the COVID-19 stressors are not significantly related to somatic complaints for people with a high amount of physical exercise (*b* = −0.06, *p* = 0.33). This research extends the cross-stressor adaptation hypothesis and provides evidence on an individual intervention of physical exercise to deal with the COVID-19 pandemic.

## Introduction

The COVID-19 pandemic has greatly influenced individual’s cognition [e.g., misperceptions, Pennycook et al. (2022)], emotion [e.g., fear, Tzur Bitan et al. (2020)], behaviors [e.g., social distancing, Carvalho et al. (2020)], health [e.g., somatic complaints, Shevlin et al. (2020)], and psychological health [e.g., depression, anxiety, and sleep disorder, Alonzi et al. (2020) and Canady (2020)]. Psychologists, educational researchers, and crisis management researchers proposed various intervention models to deal with the COVID-19 pandemic such as the cognitive model (Beck, 1993), critical incidence stress management (Carleton et al., 2020), and cultivating community resilience (Frounfelker et al., 2020). However, these models and ways for intervention largely depend on psychological aids and management from organizations or support from other individuals. How can individuals in group crisis help themselves? Physical exercise, especially exercises during quarantine and self-isolation, is recommended as a good personal tactic to overcome the negative effects such as sleep disorders and psychological problems during the pandemic (Bentlage et al., 2020; Chtourou et al., 2020), which achieved support from initial empirical evidence (e.g., Trabelsi et al., 2021a). Physical exercise refers to physical activities that help increase the heart rate, and usually bring on a sweat (U.S. Department of Health and Human Services, 2008). Generally, physical exercise or physical fitness can be regarded as a significant health-related marker (Ortega et al., 2008) and it is beneficial for psychological health such as reducing anxiety (Williams et al., 2016), stress (Tong et al., 2021a), burnout (Toker and Biron, 2012), and promoting sleep (Gebhart et al., 2011). Although the frequency of physical activity is reduced during the pandemic (Ammar et al., 2020a; Trabelsi et al., 2021a), physical exercise may be an inexpensive and accessible means to deal with the COVID-19 stressors.

### Theoretical Background

Based on the cross-stressor adaptation hypothesis (Sothmann et al., 1996), exercise can build up an integrated physiological system, induce adaptation to stressed exercise situations, and generalize the above prepared system to non-exercise adaptations. Some research established the validity of this cross-stressor adaptation hypothesis. For example, the stress-buffering effect of physical exercise was supported by evidence from the hypothalamus-pituitary-adrenal (HPA) axis (Zschucke et al., 2015), evidence from physiological responses [e.g., heart rate and salivary free cortisol, see Klaperski et al. (2013) and Ensari et al. (2020); heart rate variability, see Von Haaren et al. (2016)] and evidence from responses in social interactions (e.g., Tong et al., 2021b). However, research had inconsistent findings that physical activity and physical fitness can (Von Haaren et al., 2016) or cannot (Gnam et al., 2019) help adaptations to a real-life mental stressor. Thus, it is important to examine whether physical exercise can reduce an individual’s physiological and psychological responses to the COVID-19 stressors and prevent mental (and further physical) health issues during the COVID-19 pandemic. Although Chauhan et al. (2015) proposed several possible mechanisms involved in cross-stressor adaptation, the physiological and psychological responses are seldom examined in one integrated model. The present research aims to explore whether and how physical exercise can help people deal with the COVID-19 stressors from both physiological and psychological perspectives.

Based on the cross-stressor adaptation hypothesis (Sothmann et al., 1996), physical exercise may have the stress-buffering effect to reduce both somatic complaints and worry complaints as responses to COVID-related stressors. First, physical exercise may help deal with the COVID-19 stressors by buffering potential physiological responses such as somatic complaints. Somatic health complaints are popularly used as outcomes for stress and indicators of well-being (e.g., Sonnentag et al., 2010; Herr et al., 2018). The COVID-19 stressors increase general somatic symptoms such as gastrointestinal and fatigue symptoms (Shevlin et al., 2020). Physical exercise can build up an integrated physiological system and generalize this prepared system to non-exercise adaptations such as adaptation in the pandemic, as the cross-stressor adaptation hypothesis (Sothmann et al., 1996) suggested. Many biological processes can be triggered within the human body by physical exercise and it helps increase the immune response to COVID-19 (e.g., Roviello et al., 2021).

Second, physical exercise may help deal with the COVID-19 stressors by buffering potential psychological responses such as worry complaints. Worry complaints are significant indicators of crisis response and well-being during the COVID-19 pandemic (e.g., Liu et al., 2020). Researchers suggested further investigation on worry, threat, or fear of the COVID-19 pandemic (Guberina and Wang, 2021). Qualitatively, researchers argued that COVID-19 stressors and corresponding economic uncertainty increase social identity disturbance, job uncertainty, and psychological well-being (Godinic et al., 2020). These lead to worry complaints. Quantitatively, the COVID-19-related stressors [e.g., being mandated to relocate, Conrad et al. (2021); COVID-19 exposed first responders, Vujanovic et al. (2021)] increase worry complaints. As the cross-stressor adaptation hypothesis (Sothmann et al., 1996) argued, physical exercise induces adaptations to stressed exercise situations, which can further generalize adaptations to non-exercise and real-life stressors. Physical exercise is a viable means to reduce the risk of psychological strain and overcome psychological problems during the pandemic (e.g., Ammar et al., 2020b; Bentlage et al., 2020; Chtourou et al., 2020).

Based on the above arguments and evidence, we propose the following hypotheses.

**Hypothesis 1.** Physical exercise moderates the relationship between the COVID-19 stressors and somatic health complaints, such that the relationship is weaker and positive when the level of physical exercise is high (vs. low).

**Hypothesis 2.** Physical exercise moderates the relationship between the COVID-19 stressors and psychological worry complaints, such that the relationship is weaker and positive when the level of physical exercise is high (vs. low).

Poor sleep quality or sleep disorder is an important well-being indicator during the COVID-19 pandemic (Hyun et al., 2021). Both somatic and psychological complaints can be significant predictors of sleep disorders. For example, somatic complaints were found as predictors for poor sleep in college students (Duffield et al., 2021), and worry complaints predict poor sleep quality in U.S. young adults (Hyun et al., 2021). Fear and worries are important and significant triggers inducing stress during the pandemic (e.g., Sahni, 2020). Worry, threat or fear of COVID-19 significantly impacts anxiety and depression both in Chinese (e.g., Khudaykulov et al., 2022) and the US adults (Obrenovic et al., 2021), which in turn leads to sleep disorders. Combined with hypotheses 1 and 2, we propose the following moderated mediation models.

**Hypothesis 3.** Physical exercise moderates the relationship between the COVID-19 stressors and sleep disorder via somatic health complaints, such that the mediation is weaker and positive when the level of physical exercise is high (vs. low).

**Hypothesis 4.** Physical exercise moderates the relationship between the COVID-19 stressors and sleep disorder via psychological worry complaints, such that the relationship is weaker and positive when the level of physical exercise is high (vs. low).

## Materials and Methods

### Participants and Procedures

A Monte Carlo simulation indicated that we need a sample of 210 participants with the power of 0.80 (α = 0.05 with an effect size of 0.20). We delivered invitations to a group of human resources professionals in a part-time training program and their friends, asking them to help recruit employees in their respective companies. Interested participants were invited to join a social media (i.e., we-chat) group for multi-wave survey links. In the first survey, participants were asked to sign consent before filling out the survey questions.

A total of 207 participants were recruited from multiple companies (e.g., high-tech, educational, insurance, commercial and trading companies) in Beijing in August 2020. Data were collected anonymously in two waves and linked by a 5-digit mobile number. The COVID-19 stressors, demographic, and control variables were collected at Wave 1, while physical exercise, somatic and worry complaints, and sleep disorder were collected at Wave 2 with an interval of 1 week to mitigate common method bias concerns. We delivered 336 surveys and received 299 with a response rate of 88.99%. We delivered 299 surveys and received 280 with a 93.65% response rate. The final sample has 207 valid data with a 61.61% response rate. Participants who failed the two attentional check items (e.g., “please answer with “Strongly Disagree”; *N* = 72) and one participant who was a confirmed case for COVID-19 were excluded from the final analyses. This is the second article from a broader data collection effort.

In the final sample, participants ranged from 20 to 66 years old with an average of 34.3 ± 8.4, with 46% female and 56% married. In terms of education level, 30% are below a bachelor, 49% are a bachelor, and 21% are above a bachelor’s degree.

### Measures

All the surveys were in Chinese. Some were developed in Chinese (e.g., COVID-19 stressors, worry complaints, physical exercise), and others were translated following a standard translation procedure (Brislin, 1970).

#### COVID-19 Stressors

Adapted from Zhao and Zhou (2020), participants were requested to answer four items to indicate the COVID-19 stressors: “whether you experienced suspected infection and took a COVID-19 test,” “whether you learned people in the same community with confirmed infection,” “whether you learned someone who you know with confirmed infection,” and “whether you went for a business trip during the pandemic.” Due to that it was the post-pandemic era after the epidemic peak and turning point emerged in many countries before August 2020, some events in the original checklist (e.g., lockdown, witnessed people dying from the infection, lacked necessities and medical care) were not popular for working adults in cities like Beijing. In this checklist, “Yes” was coded as 1 while “No” was coded as 0 for each item. The average score indicated indexes of the COVID-19 stressors ranging from 0 to 1.

#### Physical Exercise

Physical exercise was rated by the three-item Physical Activity Rank Scale-3 (Liang and Liu, 1994). Participants were requested to self-report physical activity in terms of intensity, duration, and frequency during the pandemic. Each item has five levels. As an example, participants were requested to rate on a frequency item: “the frequency of your physical exercise during the pandemic is, (A) less than one time per month, (B) 2∼3 times per month, (C) 1∼2 times per week, (D) 3∼6 times per week, and (E) one time every day.” The total physical activity equals “intensity × (duration-1) × frequency,” ranging from 0 to 100. Similar measures were popularly used in psychological research (e.g., Toker and Biron, 2012).

#### Somatic Complaints

Participants reported their somatic complaints during the pandemic using a symptom list (α = 0.76) adapted from the established subjective health complaints scales (e.g., Eriksen et al., 1999; Herr et al., 2018), including “headache, stomach pain, abdominal pain, breathing difficulties, neck/back pain, skeletal muscular pain, and tiredness.” Participants responded with a 5-point Likert scale ranging from *never/seldom* (1) to *nearly daily* (5).

#### Worry Complaints

Participants reported their worry complaints on a six-item (α = 0.87) COVID-19 pandemic-related worry scale (Liu et al., 2020), ranging from *strongly disagree* (1) to *strongly agree* (7). A sample item was “I am worried about keeping in touch with loved ones during social distancing protocols.”

#### Sleep Disorders

We used the seven-item (α = 0.90) Karolinska Sleep Questionnaire (Kecklund and Åkerstedt, 1992; Sample items: “difficulties falling asleep,” and “disturbed sleep”), ranging from *never* (1) to *always/daily* (7).

#### Controls

Due to the impact of personal pandemic risk perception on individual psychology (Kim et al., 2016), risk perception was controlled together with demographic variables such as age, gender, education, and marital status in the present research. We used Kim et al.’s (2016) scale (α = 0.82) to measure personal risk perception (Sample item: “I feel vulnerable to COVID-19 infection”), using a scale ranging from *never* (1) to *always/daily* (7).

### Statistical Analysis

The hypothesized moderated mediation model was tested by the Process Macro for SPSS (i.e., Model 7). The COVID-19 stressors and physical exercise were standardized before regression analyses. The COVID-19 stressors were included as the independent variable, physical exercise as the moderator, and sleep disorder as the dependent variable. Both somatic and worry complaints were included as potential mediators in the research model.

## Results

Descriptive statistics and correlations for all variables are shown in Table 1. A confirmatory factor analysis confirmed the focal measurement model (i.e., somatic complaints, worry complaints, and sleep disorder; item parceling for seven-item variables) was acceptable: χ^2^ = 168.24, *df* = 51, *RMSEA* = 0.11, *CFI* = 0.92, *SRMR* = 0.08. It is better than alternative models combining any two focal variables, 102.47 ≤Δχ^2^(Δ*df* = 2) ≤ 499.01, indicating good discriminant validity.

**TABLE 1:** Descriptive statistics and intercorrelations of study variables.

Variable	1	2	3	4	5	6	7	8	9	10
1 Age	−									
2 Gender	0.23**	−								
3 Education	0.07	0.11	−							
4 Marital status	0.56**	0.20**	0.11	−						
5 Personal-risk perception	0.11	0.06	0.09	0.12	−					
6 The COVID-19 stressors	0.06	–0.08	0.01	–0.03	0.02	−				
7 Physical exercise	–0.12	−0.25**	–0.08	−0.17*	–0.12	–0.01	−			
8 Somatic complaints	–0.12	–0.04	0.07	–0.13	0.06	0.08	–0.003	−		
9 Worry complaints	–0.07	0.03	0.06	0.004	0.27**	–0.01	–0.08	0.16*	−	
10 Sleep disorder	–0.08	–0.05	0.17*	–0.10	0.30**	–0.001	–0.11	0.50**	0.39**	−
*N*	207	207	207	207	207	207	207	207	207	207
*M*	33.79	1.46	1.90	0.56	2.46	0.32	22.64	1.51	2.56	2.64
SD	8.07	0.50	0.71	0.50	1.13	0.19	23.64	0.59	1.23	1.28

*p < 0.05. **p < 0.01. Gender (1 = male, 2 = female); Education (1 = below bachelor, 2 = bachelor, 3 = above bachelor); Marriage status (1 = married, 2 = unmarried).

**FIGURE 1:**
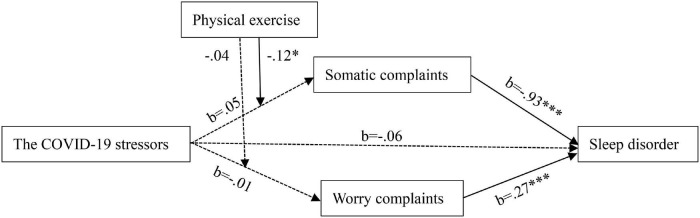
Proposed moderated mediation model. Dotted arrows represent the effects that were included in our hypothesis but turned out not significant in the analysis. ^∗^*p* < 0.05. ^∗∗∗^*p* < 0.001.

**FIGURE 2:**
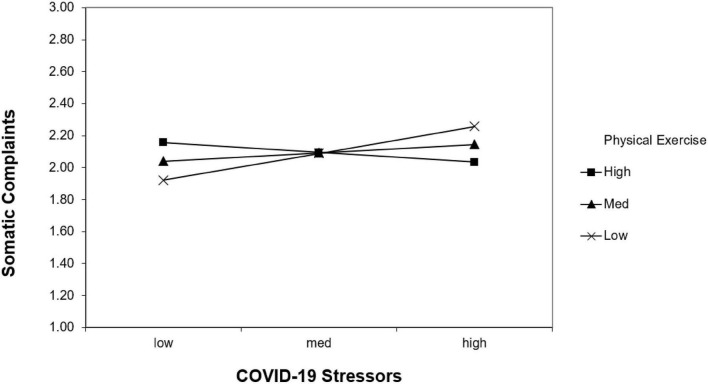
Interaction effect of the COVID-19 stressors and physical exercise on somatic complaints.

We used structural regression and simple slope analyses to test our hypothesized moderated mediation model, using standardized independent (i.e., the COVID-19 stressor) and moderator variable (i.e., physical exercise) to predict the important well-being outcome (i.e., sleep disorder). As shown in Figures 1, 2 and Table 2, the interaction between the COVID-19 stressors and physical exercise significantly predicted somatic complaints (*b* = −0.12, *p* = 0.01) rather than worry complaints (*b* = −0.04, *p* = 0.06). It supported Hypothesis 1 but failed to support Hypothesis 2. Simple slope analyses indicated that the relationship between the COVID-19 stressors and somatic complaints was positive for people who are achieving less physical exercise (-1 SD below the sample mean; *b* = 0.17, *z* = 0.06, *t* = 2.66, *p* = 0.01), but not for those who are achieving more physical exercise (+1 SD above the sample mean; *b* = −0.06, *z* = 0.06, *t* =−0.98, *p* = 0.33).

**TABLE 2:** Moderated mediation analysis.

Mediation and Dependent variable model									
Variable	Outcome: Somatic complaints			Outcome: Worry complaints			Outcome: Sleep disorder		
			
	*B*	SE	*p*	*B*	SE	*p*	*B*	SE	*p*
1 Age	−0.01	0.01	0.32	−0.02	0.01	0.15	0.01	0.01	0.67
2 Gender	0.01	0.09	0.89	0.04	0.18	0.82	−0.15	0.15	0.30
3 Education	0.07	0.06	0.22	0.06	0.12	0.61	0.21*	0.10	0.04
4 Marital status	−0.09	0.10	0.39	0.06	0.21	0.76	−0.24	0.46	0.17
5 Personal-risk perception	0.04	0.04	0.35	0.30***	0.08	<0.001	0.23***	0.07	<0.001
6 CS (The COVID-19 stressors)	0.05	0.04	0.21	−0.01	0.08	0.92	−0.06	0.07	0.41
7 PE (Physical exercise)	−0.01	0.04	0.89	−0.07	0.09	0.45	–	—	—
8 Interaction CS × PE	−0.12*	0.04	0.01	−0.04	0.09	0.63	—	—	—
9 SC (Somatic complaints)	—	—	—	—	—	—	0.93***	0.12	<0.001
10 WC (Worry complaints)	—	—	—	—	—	—	0.27***	0.06	<0.001

**Moderated mediation effects predicting sleep disorder with PE as moderator**									

**IV**	**Mediator: Somatic complaints**					**Mediator: Worry complaints**			
				
	**Index**	**95% CI *−LL***	**95% CI *−UL***			**Index**	**95% CI *−LL***	**95% CI*−UL***	

CS	−0.107*	**−0.224**	**−0.009**			−0.012	−0.074	0.044	

**Conditional indirect effects for moderated mediations predicting sleep disorder at PE = ± 1 SD**									

**IV**	**Mediator**	**Moderator**		**Indirect effect**		**95% CI-LL**	**95% CI-UL**		

CS	SC	−1 *SD*		**0.151**		**0.014**	**0.284**		
		0 (Mean)		0.047		−0.039	0.135		
		+ 1 *SD*		−0.063		−0.220	0.061		
CS	WC	−1 *SD*		0.009		−0.067	0.090		
		0 (Mean)		−0.002		−0.056	0.042		
		+ 1 *SD*		−0.014		−0.094	0.055		

IV, independent variable; CI, confidence interval; LL, lower limit; UL, upper limit. Values in bold type indicate that the CI excludes zero. *p < 0.05. ***p < 0.001.

The moderated mediation index was significant in predicting sleep disorder via somatic complaints (*index* = −0.11, *SE* = 0.06, 95% *CI* [−0.22, −0.01]) but not significant via worry complaints (*index* = −0.01, *SE* = 0.03, 95% *CI* [−0.07, 0.04]). It supported Hypothesis 3 but failed to support Hypothesis 4. Specifically, the indirect effect relating the COVID-19 stressors to sleep disorder via somatic complaints was significant for people who are achieving less physical exercise (−1 SD below the sample mean; *effect* = 0.15, *z* = 0.07, 95% *CI* [0.01, 0.28]), but not for those who are achieving more physical exercise (+1 SD above the sample mean; *effect* = −0.06, *z* = 0.07, 95% *CI* [−0.22, 0.06]).

## Discussion

This research explores the stress-buffering benefit of physical exercise on the COVID-19 stressors, in terms of both physiological and psychological responses. When the level of physical exercise is high, somatic complaints rather than worry complaints were buffered. This finding is consistent with the established stress-buffering effect on physiological responses (e.g., Klaperski et al., 2013; Von Haaren et al., 2016; Ensari et al., 2020). Although both physical exercise and sleep patterns were deleteriously affected by the pandemic (e.g., Trabelsi et al., 2021b), physical exercise is still a helpful way to buffer the effect of COVID-19 stressors on sleep disorders via physiological responses. Physical exercise is not only necessary to overcome the negative effects associated with the reduction of movement and activities during the pandemic (Bentlage et al., 2020; Chtourou et al., 2020), but also effective in dealing with experienced risks in other COVID-related stressors such as suspected infection and business trips during the pandemic.

Although previous research provided some psychological evidence on the positive cross- stressor adaptation between exercise and psychosocial or interpersonal stress (e.g., Tong et al., 2021b), our findings showed that we need more evidence to facilitate and validate this psychological adaptation process. Worry complaints (in the present research) or affective responses (Klaperski et al., 2013) may be not significant indicators for psychological adaptation. Rather, physical exercise may cultivate other psychological resources to buffer the COVID-19 stressors. For example, physical exercise may function as a behavioral distraction that disengages the individual mentally from the stressful situation (e.g., Altshuler and Ruble, 1989). It may achieve recovery by allowing people to replenish the resources and energy (e.g., Sonnentag and Zijlstra, 2006). Physical exercise may also improve individual self-efficacy and mastery (e.g., Salmon, 2001). Physical exercise delivered by technology-based solutions may promote social inclusion via virtual groups (Ammar et al., 2020b). All the above cognitive, energy, self-related, and interpersonal psychological resources may be examined, besides affective responses, as important mechanisms in future research to better understand the cross-stressor adaptation process.

The originality of the current research is to examine the stress-buffering effect of physical exercise in dealing with significant real-life stressors based on the cross-stressor adaptation hypothesis (Sothmann et al., 1996). The present research contributes to the literature in two ways. First, it extends the cross-stressor adaptation hypothesis (Sothmann et al., 1996) by comparing the effect of the physiological and psychological responses in one integrated model. The potential differences between physiological and psychological responses may help explain why the research findings are inconsistent when exploring the cross-stressor adaptations in real-life situations (e.g., Von Haaren et al., 2016; Gnam et al., 2019). Examining the detailed responses and mechanisms would help people achieve a better knowledge of cross-stressor adaptation. Second, it extends the positive cross-stressor adaptation from exercise-psychosocial stress (Chauhan et al., 2015) to exercise-pandemic stress. Future research would explore cross-stressor adaptations between multiple categories.

Practically, our research supports the importance of an individual intervention of physical exercise during the COVID-19 pandemic. To deal with the unhealthy lifestyle associated with the pandemic, physical exercise interventions can be delivered to the general population via fitness apps or online chat groups (e.g., Ammar et al., 2020c). These technology-based interventions and social media communications were proved to be convenient and helpful to maintain the quality of life (Guo et al., 2020). Relying on physical exercise, people may have an alternative and inexpensive means to deal with the COVID-19 stressors.

The present research also has some limitations. First, although the stressors and other variables were measured at different waves, all the variables were self-reported. Future research may use objective indicators or peer ratings to better deal with common method bias. Second, both the mediators and the outcome variable were collected at Wave 2. These observation data provided initial support for the stress-buffering effect of physical exercise. But future research can use experiments to further validate the physiological and psychological responses as potential important mechanisms. For example, Bo et al. (2019) proved the cognitive function of physical exercise with a randomized controlled trial. Third, similar pandemic responses were found across cultures (e.g., Obrenovic et al., 2021; Khudaykulov et al., 2022), but Chinese people present distress more somatically and the Westerns present distress more psychologically (e.g., Ryder et al., 2008), so whether our findings can generalize to people from other countries needs to be examined.

Further, future research should examine more response-related variables to do a better comparison between psychological and physiological responses to non-exercise stressors. Although worry or fear of COVID-19 is regarded as important and warrants further investigation for the pandemic (e.g., Guberina and Wang, 2021; Obrenovic et al., 2021; Khudaykulov et al., 2022), we found that physical exercise is more important to buffer physiological (e.g., somatic complaints) rather than psychological responses (e.g., worry complaints). However, physical exercise may be helpful to adjust other psychological responses to COVID stressors. For example, physical exercise intervention delivered by technology-based solutions such as fitness apps or chat groups (e.g., Ammar et al., 2020c) may help promote social inclusion and reduce the risk of psychosocial strain (Ammar et al., 2020b).

## Conclusion

Physical exercise can help deal with the COVID-19 stressors by reducing somatic complaints. Comparatively, the stress-buffering effect of physical exercise on sleep disorder is less for psychological affective responses such as worry complaints. People may use physical exercise interventions to deal with the negative effects of COVID-19 stressors.

## Data Availability Statement

The raw data supporting the conclusions of this article will be made available by the authors, without undue reservation.

## Ethics Statement

The studies involving human participants were reviewed and approved by School of Psychological and Cognitive Sciences, Peking University (#2020-06-06). Written informed consent for participation was not required for this study in accordance with the national legislation and the institutional requirements.

## Author Contributions

JQ and JT contributed to the study conception and design. JT wrote the first draft of the manuscript. All authors performed the material preparation and data collection and analysis, commented on previous versions of the manuscript, and read and approved the final manuscript.

## Conflict of Interest

The authors declare that the research was conducted in the absence of any commercial or financial relationships that could be construed as a potential conflict of interest.

## Publisher’s Note

All claims expressed in this article are solely those of the authors and do not necessarily represent those of their affiliated organizations, or those of the publisher, the editors and the reviewers. Any product that may be evaluated in this article, or claim that may be made by its manufacturer, is not guaranteed or endorsed by the publisher.
